# Stakeholder relationships and corporate social goal orientation: Implications for entrepreneurial psychology

**DOI:** 10.3389/fpsyg.2022.942294

**Published:** 2022-10-28

**Authors:** Xiaowei Lu, Ya Sheng, Yao Xiao, Wei Wang

**Affiliations:** ^1^College of Economics and Management, Zhejiang A&F University, Hangzhou, China; ^2^School of Business Administration, Zhejiang Gongshang University, Hangzhou, China; ^3^School of International Studies, Zhejiang Business College, Hangzhou, China

**Keywords:** social goal orientation, Chinese, entrepreneurs’ psychology, stakeholder relationships, economic-social framework

## Abstract

As the sensitivity to corporate social responsibility (CSR) continues to grow, the goal of enterprises has expanded beyond the sole pursuit of economic value. Corporate social goal orientation has therefore come to occupy a central position in entrepreneurs’ psychology and the transition away from a market-only economy. This study uses secondary data from 4,288 samples of 725 Chinese-listed companies from 2009 to 2020 to explore the driving factors in social goal orientation based on the characteristics of sample companies and their industry groups from the perspective of stakeholder relationships. The results can be summarized as follows: (1) there is an inverted U-shaped relationship between government stakeholder relationships and social goal orientation, and there is a significant positive relationship between financial stakeholder relationships, market stakeholder relationships, and corporate social goal orientation. (2) The correlation between single-dual stakeholder relationships and social goal orientation is not consistent. In light of the nature of the roles of government and the market, the correlation between the government–market dual relationship and corporate social goal orientation is not significant. However, there is a significant correlation between the finance–government dual stakeholder relationship and social goal orientation; that is, the dual stakeholder relationship maintains the existence of non-institutional capital and corporate financial capital. Moreover, there is no significant correlation between the market–finance dual relationship and corporate social goal orientation, and there is substitutability between market and financial stakeholder relationships. With the deepening of our understanding of CSR, the core goal of enterprises is no longer confined to the pursuit of economic value, and their social goal orientation has come to be regarded as a major driving force in sustainable development. This study enriches the research on the relationship between stakeholder relationships and shows that stakeholder relationships also have important significance to both achieving corporate goals and shaping entrepreneurs’ psychology.

## Introduction

As China’s economic transformation begins to reach maturity, the wealth of **Chinese** enterprises has increased exponentially, and sustainable development must effectively address non-wealth-related social problems. For example, environmental awareness has been a concern of the global main agenda (Barba-Sánchez et al., 2022). Three of the 17 sustainable development goals for 2030 signed by the United Nations are related to social issues such as environment (Barba-Sánchez et al., 2022). Pursuing these opportunities, respecting environmental protection, social employment and pension insurance, and creating responsible products, processes and services have led to social goal-oriented enterprises with a high sense of social responsibility. The case of “Teach for America” (Thomas and Mockler, 2018) demonstrates the importance of corporate social orientation, which not only solves the problem of educational inequality and identifies the corresponding social responsibilities, but also promotes the reform of corporate socialization. As the ultimate goal of enterprise socialization, scholars believe that enterprise social goal orientation (**May et al., 2021**) ultimately lies in the degree to which society benefits from enterprises’ activity. Enterprises are formed by the coexistence and cooperation of multiple subjects, so their enterprise social goal orientation is inseparable from and dependent on each subject in the external system. In essence, the relationship between stakeholders and enterprises is an asymmetric exchange of resources (Pajunen, 2006). Different stakeholders will dynamically choose influence and control strategies according to the degree of the enterprises’ dependence on their resources (Sheng and Li, 2016), and then promote the socialization of enterprises according to the differences in resource allocation. The consideration of multi-subject and multi-type cooperation between enterprises and stakeholders is a beneficial contribution of the existing research on social goal orientation.

Some scholars have recognized the important role of stakeholder relationships in the realization of enterprise value, in the theoretical analysis, it is found that entrepreneurs who are aware of social responsibility seek stakeholder relationships (Barba-Sánchez et al., 2021) and maintain a positive relationship quality with core stakeholders such as society, which is conducive to solving social problems such as environmental protection and employment. However, the existing research on stakeholder relationships takes a theoretical view that does not consider how such relationships function in practice. The prior studies on stakeholder relationships (e.g., Jiang et al., 2020) demonstrate the following two deficiencies. First, the previous studies regard solving social problems as a matter that falls within the government’s duties. However, corporate social development is not a “government-centric” governance model but rather plays a role through their open systems, so whether the driving force in social goal orientation shifts from the government to society at large and market-oriented stakeholder relationships has not yet been addressed by the existing research. Second, previous studies (e.g., Sheng, 2020; Lu and Sheng, 2021) tend to take enterprises and stakeholders as single subjects, but the research based on relationships does not focus on either of the two parties but rather on the structure, form, and process of the interaction between them. The establishment of social decision-making and response mechanisms in the context of stakeholder relationships is the key to sustainable development. Finally, sustainability depends on the environment. Freeman et al.’s (2010) research has inspired scholars to discuss the interaction between enterprises and the external environment. It is inevitable that heterogeneous environmental contexts will influence and, to a certain extent, control enterprises’ behaviors, and ultimately determine their value (Sauerwald and Peng, 2013). However, no prior studies have investigated this key relationship.

Based on this, we conduct a literature review and theoretical study (Bin et al., 2020; May et al., 2021; Paruzel et al., 2021) to investigate A-share-listed companies using a sample of society-oriented enterprises by leveraging the existing studies on the relationship between stakeholders and corporate social goal orientation (Farmaki, 2019). The impact of stakeholder relationships on the corporate social goal orientation was firstly identified; whether this impact differs depending on the relationship type was then investigated. Stakeholder relationships are classified according to their social goal orientation. A high level of social responsibility encourages entrepreneurs to take a positive attitude, cooperate deeply with external core stakeholders, and promote the realization of the final economic and social results (Barba-Sánchez et al., 2021). Thus, addressing the problems in stakeholder relations is of great significance for entrepreneurs’ psychology, decision-making, and enterprise socialization in general. The results of this study show that the relationship between different types of stakeholders and enterprises’ social goal orientation is heterogeneous. Moreover, the relationship between stakeholder relationships and corporate social goal orientation also changes according to the degree to which different subsystems are integrated. The results of this research hold true after a series of robustness tests.

Unlike the previous studies which once regarded solving social problems as a matter within the government’s responsibility, the specific contribution of this study lies in these aspects: First, corporate social development is not a “government-centered” governance model but plays a role through the open system in which it is located. Based on this, this study provides evidence support for the shift of the driving force of social goal orientation to the relationship between social and market-related stakeholders. Second, previous studies often focused on a single subject among enterprises or stakeholders. However, studies based on relationship did not focus on either party, but on the structure, form and process of interaction between the two parties. The establishment of a decision-making mechanism and a sensitive response mechanism for social needs under the relationship between stakeholders is the key to the sustainable development of enterprises. The study provides evidence support for the role of stakeholder relations from the perspective of enterprises’ response to external social needs. Finally, the sustainability of corporate interests must depend on the environment. Heterogeneous environmental situations cannot be ignored to influence and control the behavior of enterprises. Based on the interactive perspective of enterprises and the external environment, this study not only provides a new reference for exploring its final strong impact on enterprise value and market exchange, but also promotes entrepreneurs to adopt a responsible attitude, carry out in-depth cooperation with external core stakeholders, and promote the realization of economic and social needs (Barba-Sánchez et al., 2021).

## Theoretical basis and literature review

### Theoretical basis

In this study, we draw upon stakeholder and corporate social responsibility (CSR) theory to examine the effect of stakeholder relationships on social goal orientation. According to stakeholder theory (Freeman, 1984; Freeman et al., 2010), stakeholder groups are concerned with the operations and bear most of the risks of the enterprise and form the source of their core competitiveness. As such, the interaction between the enterprise and its external subjects is the basis of the stakeholder relationship research (Charles et al., 1992; Lin, 2010). Mitchell et al. (1997) believe that the most important stakeholder attribute is their resources. Stakeholders have the resources necessary for the survival and development of the enterprise, which means that the parties to the relationship must sacrifice their individualism and rely on each other through the process of resource allocation. At the same time, they must communicate with each other, reach common understandings, and take joint actions (Sheng, 2020) to realize shared value. For example, Bridoux and Stoelhorst (2016) identify that stakeholder relationships can contribute to value creation, especially when they are not driven by self-interest. Amis et al. (2020) believe that relationship governance is the key factor in enterprises continuously obtaining scarce resources in the stakeholder network, which can help enterprises improve the efficiency of their resource allocation. Strand and Freeman (2015) also believe that by pursuing strong stakeholder relationships, enterprises are able to implement effective value creation strategies (Jiang et al., 2020; Wang et al., 2022). In other words, considering stakeholder relationships as a business strategy has become an important way for Chinese enterprises to gain a competitive advantage (Sheng and Yu, 2018).

According to CSR theory, social responsibility is the cornerstone of social goals. Bowen (1953) first proposed that enterprises should assume social responsibility in addition to being responsible to shareholders (Gao et al., 2020). Most of the existing studies (e.g., Luo et al., 2015; Hahn et al., 2016) show that corporate social goal orientation brings business expertise and new technologies to the non-profit sector in order to help it achieve social goals. In essence, however, social goal orientation is a mixed model which can seek to solve social problems while improving efficiency by adopting commercial management techniques to obtain maximum benefit. Social goal orientation is in line with the characteristics of both for profit and non-profit corporate activity (Zarzycka et al., 2021). Finally, while creating profits and remaining accountable to shareholders and employees, enterprises must also meet the needs of external stakeholders. At the same time, CSR requires enterprises to go beyond the traditional concept of profitability and emphasize the importance of social value in terms of the environment and society at large (Zheng et al., 2014).

### Literature review

The integration of stakeholders into enterprise development is both realistic and currently in high demand. Enterprises that ignore external stakeholders in their decision-making are likely to suffer in terms of their sustainable development; in other words, there are likely to be disagreements regarding whether there are economic benefits of social responsibility (Crişan-Mitra et al., 2020; Vǎtǎmǎnescu et al., 2021). Corporate social value is interwoven with non-economic motivations such as government connections, government subsidies, and social costs. Therefore, almost all corporate decisions and behaviors will inevitably be influenced by stakeholder groups in the external system. The realization of sustainable development involves rethinking external social motivations. In addition, previous research has focused on the relationship between stakeholders and corporate strategy in general (Bridoux and Stoelhorst, 2016), that between stakeholders and board decision-making, and that between stakeholders and evaluating managers’ performance. He et al. (2019) verify that the relationship between enterprises and banks could enable them to obtain a lower cost of capital and favorable terms. Because of the social embeddedness of business relationships in China, relationships are ubiquitous, which improves efficiency and forms an important informal governance mechanism that creates social and economic value in China. Therefore, in the Chinese context, it is critical to learn how to cultivate and maintain stakeholder relationships. The paper is based on the theory of stakeholder management and regards the enterprise and its external subjects as stakeholders (Lin, 2010). Members of the relationship network must sacrifice their individualism, respectfully communicate with each other, reach common understandings, and take joint actions (Li and Sheng, 2018) to ensure that their shared values are upheld.

Corporations are increasingly interested in socially oriented innovation (Wang, 2021). A growing number of studies (e.g., Farmaki, 2019; Lǎzǎroiu et al., 2020) have also realized that the link between corporate sustainability and social programs plays a central role in social goal orientation (OECD, 2015) and reveal that stakeholder-embedded corporate development conforms to both theory and practice. “Embeddedness” theory points out that any corporation that operates in a social structure and exchanges resources with other organizations in a relationship network forms “social capital,” and the effectiveness of that social capital depends not only on its network structure but also on the relationships themselves (Granovetter, 1985). Corporate economic and social behaviors are complex and inevitably affected by the relationships between, external cooperation with, and competition among stakeholder groups in the social system (Granovetter, 1985). Bridoux and Stoelhorst (2016) point out that stakeholder relationships can contribute to value creation, especially when they are not driven by self-interest. By pursuing stakeholder relationships, enterprises seek to create value (Strand and Freeman, 2015). However, Li and Sheng (2018) point out that, whether from the perspective of the enterprise or the stakeholders, most studies ignore the characteristics of the relationship between stakeholders and enterprises and therefore cannot fully explain their importance. In light of the fact that enterprises and stakeholders are closely linked, that private enterprises in China are undergoing a transformation from economic to social governance, and that scholars have not yet conducted an in-depth study on how stakeholders participate in the process of corporate socialization through relationships (Razmus and Laguna, 2018; Sheng, 2020), it is logical to discuss corporate social goal orientation from the perspective of stakeholder relationships, which forms the theoretical basis of this study.

## Research design and hypotheses development

### Research design based on an economic–social framework and stakeholder identification

#### The economic–social framework

In this study, we explore the influence of stakeholder relationships on the basis of an economic–social framework. On the one hand, there are significant differences in the interaction modes and processes between enterprises and stakeholders due to the diversity of stakeholders. Therefore, it is necessary to find the basic elements that explain these stakeholder relationships. On the other hand, Hill and Jones (1992) emphasize the importance of relationships at the economic level, while Friedman and Miles (2002) emphasize the social and cultural aspects at the institutional level. However, emerging interdisciplinary research integrates the related economic theory with sociology to explain network interactions in a comprehensive way.

Granovetter (2018) points out that the actions and behaviors of actors in a social system indicate that they are participating in a social relationship. This means that to analyze the behaviors of social actors, the embedded system of relationships must be considered because economic and social elements work together to shape the behaviors of social actors, and thus a more comprehensive explanation of those behaviors can be made by considering both factors at the same time. The social actors in Granovetter’s (1985) embeddedness theory include both individual and group actors that are organized in various ways (e.g., enterprises and communities), while those analyzed in this study are enterprises and their stakeholders. According to embeddedness theory, in a relationship composed of two actors, there are multiple interactions of different dimensions, including the social and economic dimensions. Both dimensions must be taken into account in order to fully explain behavior. Therefore, the application of the economic–social framework can more comprehensively analyze the multiple dimensions of stakeholder relationships, thus providing a foundation for stakeholder management theory from the perspective of relationship formation and maintenance. In this study, we discuss the economic and social dimensions of stakeholder relationships from the perspective of economic sociology and focus on the significance of the social dimension to the management of corporate stakeholders.

#### Subject identification

**FIGURE 1:**
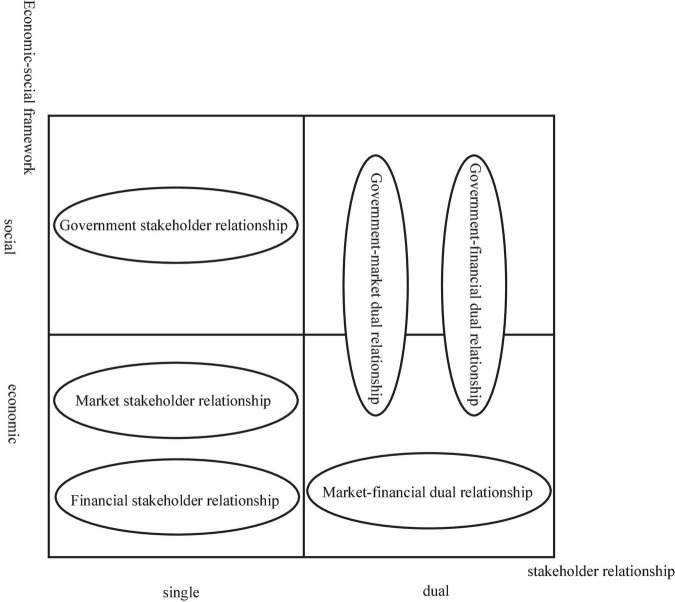
Single–dual stakeholder relationship of the economic–social framework.

Social systems are regarded as a community of economic and social actors that interact with each other to influence behavior. Therefore, there are not only economic exchanges but also social exchanges between enterprises and stakeholders (Freeman et al., 2010; Lu et al., 2020). Therefore, although the survival and development of a corporation cannot be separated from the support of its stakeholders, it cannot simply take all stakeholders as a single entity (Lin et al., 2010). In this study, we adopt Tian’s (2019) perspective of the external environment and the main external stakeholders are identified based on the identification of the external political and economic system following Clarkson (1995). First, we consider stakeholders who have economic relationships, including banks and financial institutions, and those who have political relationships, including the government (Freeman et al., 2010). We then focus on the external political and economic systems, and identify the stakeholder groups that have a core influence on corporate social goal orientation. At the same time, as the external system of the company is open, the stakeholder relationships fluctuate, so the enterprise can seek the resources needed through the heterogeneity of the stakeholder relationships (Sheng, 2020). Kujala et al. (2022) point out that the norms formed by the whole stakeholder group may be inconsistent with the demands of individual stakeholders. Considering the characteristics of enterprises and the differences in the effectiveness of various stakeholder relationships, we assume that stakeholder relationships are divided into single (e.g., government, finance, or market-based) and dual (e.g., government–market, government–finance, and market–finance) stakeholder relationships and empirically test them on this basis (see Figure 1).

### Hypothesis on the correlation between single-stakeholder relationships and corporate social goal orientation

#### Government stakeholder relationships and corporate social goal orientation

The increasing prevalence of the social concept promotes social-oriented action. However, from the perspective of the resource-based view, corporate social goal orientation is mainly constrained by two aspects: internal resource constraints and uncertainty constraints (Piotroski et al., 2015; Jeong et al., 2021). With respect to the first aspect, corporates’ social goal orientation diverts part of their operating income into social projects. However, limited resources may weaken such social goal orientation. With respect to the second aspect, the ultimate goal of enterprises is survival. Social orientation not only solves social problems but also assures that enterprises are profitable (Cai et al., 2018). However, the social strategy model is difficult to implement because it lacks legitimacy, and the currently weak institutional environment is a potential threat. Government stakeholder relationships (hereinafter referred to as “government relationships”) can effectively reduce these two constraints (Piotroski et al., 2015; Jeong et al., 2021). First, China’s historical and cultural traditions, cultural customs, and legal origins are different from those of other countries (Hao et al., 2015). Government relationships are an extension of the official economy, which has a profound historical foundation in China (Luo and Ying, 2014). As informal institutional capital owned by enterprises, government relationships are conducive to securing access to key government resources, financing, and institutional support (Bayraktar and Moreno-Dodson, 2015; Habib et al., 2017). Aldhamari et al. (2020), Xu and Yan (2020), and Jiang et al. (2021) have shown that enterprises with a government relationship are more likely to engage in business and social activity. Therefore, the innovation and efficiency of enterprises with a government relationship are better than those without a government relationship. Second, a government relationship can facilitate access to market information, resources, and policy support (Saeed et al., 2016; Yang and Tang, 2020), which improve the ability to anticipate market trends, access resources, and navigate an uncertain external environment (Razmus and Laguna, 2018). At the same time, enterprises’ sustainability and social contributions should be improved (Carroll, 2021). However, there is a paradox in the existing research; that is, if the government relationships are too close, they are more likely to induce opportunism and rent-seeking behavior, thus violating the market system and negatively impacting corporate value (Thomas and Mockler, 2018). Based on this, it is reasonable to assume that government relationships have intrinsic value and can improve corporate social goal orientation. However, relatively speaking, government relationships do not show a long-term linear increase in the promotion of social goal orientation, and the correlation between the two may have a certain threshold that maximizes its utility. Based on this, we propose hypothesis 1:

**H1:** The relationship between government stakeholders and corporate social goal orientation follows an inverted U-shaped relationship.

#### Financial stakeholder relationships and corporate social goal orientation

In view of the fact that the social role of enterprises requires financial resources, banks and other financial institutions play an indispensable role in social goal-oriented activity (Farmaki, 2019). At the same time, enterprises integrate social problems into their business to benefit both their business and society at large (Jones et al., 2018). Chinese enterprises have faced financing constraints in recent years as a result of a monopolized financial market dominated by state-owned banks, ownership discrimination, interest rate control, financial privileges, the initial public offering approval system, etc., which makes the bank–corporate relationship dynamic particularly intense. Banking relationships can help enterprises to gain more external capital at a lower cost (Kovacova et al., 2018), which is an important source of funding for social innovation projects. Corporations with such stakeholder relationships have advantages in terms of obtaining financial resources, which influences their social goal-oriented activity and efficiency. Financial stakeholder relationships (hereinafter referred to as “financial relationships”) can also reduce social goal-oriented risks. It is unlikely that enterprises will abandon social-oriented activity due to risk aversion. The close relationship between enterprises and financial institutions provides financial guarantees for the resources needed to achieve social goals (Braggion, 2011). The goal of this study is therefore to explore the influence of financing and resource allocation on social goal orientation by studying the relationship between enterprises and their financial stakeholders. We therefore assert:

**H2:** Financial stakeholders have a positive impact on corporate social goal orientation.

#### Market stakeholder relationships and social goal orientation

Market stakeholder relationships (hereinafter referred to as “market relationships”) mainly refer to relationships between enterprises and suppliers, distributors, final customers, and competitors (Park and Luo, 2001; Cui et al., 2021), which influences corporates’ social goal orientation from the aspects of market information, legitimacy, and external resources. First, the relationship between enterprises and other market entities provides enterprises with a diversity of valuable information; that is, market relationships have the function of communicating and sharing information. Such information plays an important role in formulating the right survival strategy and identifying investment opportunities (Sergiy et al., 2021). Second, market relationships can significantly reduce information asymmetries (Barney, 2018) so that enterprises can better understand the risks of socially oriented projects, thus reducing the opportunity cost of investment and improving investment returns. Finally, in the Chinese institutional context, the market uses the “invisible hand” to regulate market participation and competition (Li et al., 2022). Therefore, establishing market relationships is an important condition for achieving social goals. On this basis, strong market relationships can make all parties in an enterprise bond together, deepen the reciprocity of obligations, and reduce waste and wait times caused by resource demands. Based on the above, we propose:

**H3:** The stronger the market stakeholder relationships, the more stable the correlation between market relationships and social goal orientation.

### Hypothesis on the relationship between dual stakeholder relationships and corporate social goal orientation

#### Government–market dual relationship and corporate social goal orientation

The deepening of market rules and the centralization of government resources have formed a dual situation in which the government and the market coexist, which has led to a stakeholder network structure in which enterprises, the government, and the market pay equal attention to each other (Torelli et al., 2020). The brand battle between Wong Lo Kat and Jia Duo Bao (Chinese beverage brands) and the debate between Nongfu Spring and the Beijing Times provide strong evidence that market and government relationships are crucial to business development. However, the research on the relationship between the government and the market is in conflict with the following two factors. (1) Many studies ignore the particularity of the coexistence of government and market relationships (Sheng et al., 2011; Aldhamari et al., 2020), and (2) the multiple effects of stakeholders on the growth of enterprises from a dynamic perspective have not yet been examined (Guo and Miller, 2010). From the perspective of strategic management, more and more enterprises regard political capital as an important way to obtain competitive advantages (Schuler, 1996; Bayraktar and Moreno-Dodson, 2015): it not only enhances the competitiveness of the enterprise but also effectively captures government resources (Lin, 2016). Political and market successes are equally important to enterprise development. In sum, in an uncertain environment, most entrepreneurs prefer to seek political safety to reduce operational uncertainty, and the government is generally willing to cooperate with mature enterprises. The Research Report on Corporate Social Responsibility of China (Huang et al., 2018) points out that the overall development of the top 300 Chinese enterprises involved in social responsibility is still in its infancy, and the government and the market thus require enterprises to pursue lean management-oriented social goals. That is, the government and the market expect enterprises to assume certain responsibilities, especially those concerning social goals, while providing them with resources. We therefore propose:

**H4:** The government–market dual relationship has a significant positive impact on corporate social goal orientation.

#### The government–finance dual relationship and corporate social goal orientation

Tian et al. (2019) believes that government relationships help enterprises obtain resources, such as financing, tax incentives, and government subsidies. At the same time, given that the financial relationships of enterprises play an important role in obtaining the scarce resources needed for operations, their financial relationships have gradually attracted the attention of scholars. Based on the background of Chinese traditional culture and the “relationship network,” individuals working in banks and other financial institutions bring much-needed resources. Therefore, such relationships serve as valuable corporate social capital by transforming individual resources into the ability of enterprises to obtain financial capital. Although the financial system is the main channel through which enterprises obtain external financing, enterprises face strong financing discrimination in a financial system dominated by state-owned banks. Barney (2018) evaluates the sources of financing in China from 1995 to 2002 and finds that the ratio of external funding is as high as 90%. In other words, when the policy uncertainty faced by enterprises increases, the cost and difficulty of obtaining external funding rise due to external financing constraints. Based on this, enterprises should stockpile cash to deal with potential uncertainties (Yu et al., 2018).

Studies have found that enterprises obtain credit by establishing government relationships (Kovacova et al., 2018; Xu and Yan, 2020). In addition, the establishment of government and financial relationships also has an “information effect” and a “resource effect”; government relations can play the role of “signal transmitters” in reducing information asymmetries (Yu and Pan, 2008; Thomas et al., 2017). The existence of a financial relationship improves the ability to obtain financial resources and reduces financing constraints (Han and Fu, 2017). This dual relationship maintains the existence of both the non-institutional and financial capital, and thereby enhances enterprise value. Therefore, when enterprises have both a financial and a government relationship, they improve their risk control and are able to realize social and economic goals. Based on this, we propose:

**H5:** The government–finance dual relationship has a significant positive correlation with the social goal orientation.

#### The market–finance dual relationship and corporate social goal orientation

The market and financial relationships may exist simultaneously. It is relatively easy for stakeholders such as banks and financial institutions to collect financial and operational information. Information spillover reduces information asymmetries between banks and enterprises, and improves external supervision and control mechanisms (Wang et al., 2022). In addition, the dual relationship between market and finance takes into account the supply chain, delivers signals to external stakeholders such as banks, and enhances corporate risk decision-making value (Sergiy et al., 2021). However, there is a paradox in this relationship. The alternative financing theory shows that when the amount of bank loans is too small to meet demand, enterprises tend to improve their market relationships to relieve their financing constraints (Cai et al., 2021), and there is substitution between the market and financial relationships. At the same time, enterprises with weak market relationships face difficulties in supply chain integration, and banks are unable to use their information advantages on basis of adverse selection and moral hazard (Shaharudin et al., 2019). Furthermore, banks are less likely to finance such enterprises. The reasons for this finding may be as follows. First, the research perspectives are inconsistent. Stakeholder relationship studies from a single perspective cannot fully explain sustainable development. Second, resource scarcity and the demand for sustainable development promote the pursuit of heterogeneous stakeholder relationships. Based on this, we propose:

**H6:** The market–finance dual relationship has a significant positive correlation with corporate social goal orientation.

## Description of variables and data

### Explained variable–corporate social goal orientation

The concept of corporate social goal orientation mainly refers to the extent to which enterprises contribute to solving social problems when carrying out commercial activities. In order to comprehensively evaluate the social goal orientation, it is necessary to establish a social goal orientation (SGO) index system. In order to overcome the deficiency of any single source, multi-source data are used to evaluate the social performance of enterprises in a triangular model. Therefore, this study selects three indicators of listed companies to evaluate corporate social goal orientation, including social responsibility-related strategy formulation (i.e., systems, rules, evaluation criteria, and main business), public welfare fund investment, and CSR index disclosure. In order to analyze the social status of listed companies, this article collects the data of all A-share listed companies from the iFinD database. Based on the selection of the above social indicators, we first check whether there is any social objective strategy formulation (SGS) in the company’s disclosed annual report. Second, we consider whether the listed company had public welfare fund investment (WFI) from 2009 to 2020. Finally, based on the Rankins CSR Ratings (RKS),^1^ the SRI of 725 listed companies from 2009 to 2020 is collected, and the sociality of listed companies is analyzed and compared based on the collected data. If the company discloses relevant social goal-oriented strategies, systems and evaluation criteria, and its main business is a social goal-oriented project, it is recorded as SGS = 1; otherwise, it is recorded as SGS = 0. If there is public welfare fund input (WFI), it is recorded as WFI = 1; otherwise, it is recorded as WFI = 0. If the social responsibility index (SRI) is below 50, it is recorded as SRI = 1; otherwise, it is recorded as SRI = 2. Finally, the three indicators are converted into a range between 0 and 1.34 according to the average ratio given the CSR reporting of listed companies in voluntary disclosures. Moreover, this study takes 9 years of research data from 2009 to 2020. Therefore, the sample data are manually matched and an empirical study is conducted using 4288 samples from 725 enterprises that meet the research criteria.

### Explanatory variable–stakeholder relationships

Based on the preceding discussion on the measurement of social relationships in previous studies (e.g., Pedrini and Ferri, 2019; Jiang et al., 2020), this study manually collects relevant data and divides stakeholder relationships into the following three categories. 1. *Financial relationships*. In view of the fact that individuals’ experience in financial institutions can promote the acquisition of funding (Zarzycka et al., 2021), in this study, *FC* = 1 is defined as a financial relationship if the CEO or any board members have ever held a position in a bank or other financial institution; otherwise, *FC* = 0. 2. *Market relationships*. If the market relationships are measured by the number of posts of the CEO or board members held in other enterprises in the industry chain, when the number of posts is greater than the sample average, *MC* = 1; otherwise, *MC* = 0. Market relationships cover the relationship network, including both upstream and downstream enterprises, competitors, partners, and other market participants. A good market relationship network helps enterprises access market information and resources, obtain stable purchasing channels as well as efficient and reliable sales channels. 3. *Government relationships*. In this study, whether the CEO and board members had political connections is used to reflect whether the enterprise has strong government relationships. Previous studies (e.g., Xu and Yan, 2020; Avotra et al., 2021) have shown that if the CEO or board members have served in government or is a deputy to the National People’s Congress or a member of the Chinese people’s political consultative conference (CPPCC), the company’s publicly disclosed data serve as a benchmark to measure its relationship with the government. According to He et al.’s (2019) study, the government relationship index is constructed. Based on this, we first manually obtain senior entrepreneurs with a political background from the annual reports of listed companies. Second, considering that government relationships at different levels greatly affect the ability of enterprises to obtain resources, this study uses relevant studies for reference and divides government relationships into the following: country, province, city, county, and township (in sequential order). We sort them individually and assign them values of 0, 1, 2, 3, 4, and 5, respectively (Bar-Joseph et al., 2012). That is, in view of China’s bureaucratic system, government officials have more power and more political and economic resources than staff (Chen C. et al., 2018; Arnoldi and Muratova, 2019). In this study, senior entrepreneurs of affiliated enterprises are rated according to their bureaucratic level: the higher the bureaucratic level, the higher the degree of correlation is. Finally, the government relationship score for each company is calculated as the sum of the scores earned by its politically connected CEO and board members (Yang et al., 2018). Given the differences in political ties between China’s listed companies, in this study, the maximum difference method is used to standardize these scores and obtain the government relationship index. To be precise, the correlation index of company *i* is defined as the difference between the correlation points obtained by company *i* and the minimum correlation points in a given year divided by the difference between the maximum and minimum correlation points in a given year in the sample.

### Control variables

1. Corporate maturity (*Mat*). When dealing with the external environment, the relationship between enterprises and external stakeholders will vary according to the resources they have. The adequacy of these resources is related to the size of the enterprise. Corporate size is an important indicator in describing the degree of resource acquisition, which determines whether a company can make behavioral choices according to its own will. The definition of corporate maturity can be measured using the age of the enterprise, the cash flows, the economic cycle, the management methods, etc. However, Pellenbarg et al. (2002) points out that “age” is the most effective way to measure corporate maturity.

2. Corporate credit evaluation (*Cre*). Funds can be maintained through bank debt. At the same time, existing studies (e.g., Nave and Ferreira, 2019; Ye et al., 2020) have determined that bank loans effectively measure the market’s rating of corporate credit, so we use the corporate debt ratio to evaluate corporate credit.

## Data source and sample size

### Data source

The samples in this study are sourced from the CSMAR and iFind databases, and cninf^2^ and specific data items are collected manually from the annual reports of all A-share listed companies from 2009 to 2020. Data are collected beginning from 2009 because this study uses the CSR index, which is only available from 2009. Considering that the CSR index is only available until 2020, our study period ends in 2020. Thus, the annual reports for 2009–2020 were downloaded and the social relational data were manually sorted according to the according to the qualifications of the CEO, board members, and other insiders. At the same time, information channels such as Google, Baidu, and other search engines were used to supplement the publicly available information. The sample data were screened according to the following principles: (1) we exclude listed companies with ST (Special Treatment) and *ST status; (2) we eliminate samples with missing data; and (3) we choose social goal-oriented enterprises. There is a slight disconnect between business innovation and the pursuit of the corporate social value. More and more enterprises are seeking activities that complement their profit maximization goals. Social goal-oriented enterprises belong to a variety of industries and involve diversified stakeholders and their innovation model and business objectives are quite different from those of traditional enterprises (as shown in Table 1). The sample enterprises in this study includes a broad cross-section of services and other industries (as shown in Table 2), which allows us to comprehensively discuss the corporate social goal orientation (Sheng, 2020; Sheng and Lu, 2020). After screening, the final sample includes 4,288 observations from 725 companies.

**TABLE 1:** Variable definitions.

Variable classification	Variable symbol	Variable name	Variable definition
Explained variable	*SGO*	Social goal orientation	Standardization of social indicators
Explanatory variable	*PC*	Government relationship	Bureaucratic hierarchy of CEOs and board members with political backgrounds
	*FC*	Financial relationship	Worked in banking and other financial sectors
	*MC*	Market relationship	The number of positions held in other corporates
Control variable	*Mat*	Corporate maturity	The date of establishment to the date of statistics
	*Cre*	Corporate credit evaluation	Debt ratio

**TABLE 2:** Classification of industry and social goal-oriented projects in the sample.

Serial number	Industry	Social innovation projects of sample corporates
1	Comprehensive	Medical care, elderly care, ecological protection, medical health
2	Manufacturing	Solar energy, new energy, health service business, industrial sewage treatment, medical treatment, green energy environmental protection, medicine and health
3	Electricity, gas, and water	Certification of emission reduction, integrated energy services, sewage treatment
4	Information technology	Education, energy conservation, and environmental protection
5	Mining	Comprehensive utilization of waste resources
6	Social services	Environmental protection, sewage treatment, recycled water business
7	Construction	Environmental protection

### Model design and variable descriptions

First, hypotheses 1–3 were tested, namely, the correlations between government, financial, and market relationships and social goal orientation were tested according the following regression:


(1) S⁢G⁢O=β⁢0+β⁢1⁢P⁢C+β⁢2⁢F⁢C+β⁢3⁢M⁢C+β⁢4⁢S⁢i⁢z⁢e+β⁢5⁢M⁢a⁢t+β⁢6⁢C⁢r⁢e+ξ

where *PC*, *FC*, and *MC* represent government, financial, and market relationships, respectively as the explanatory variables. *SGO* represents social goal orientation as the explained variable. Other variables in the model are control variables that may affect social goal orientation. In order to test hypotheses 4–6, models 2–4 are set up to test the impact of dual stakeholder relationships on social goal orientation.


(2) S⁢G⁢O=a⁢0+a⁢1⁢P⁢C+a⁢2⁢S⁢i⁢z⁢e+a⁢3⁢M⁢a⁢t+a⁢4⁢C⁢r⁢e+a⁢5⁢F⁢C×P⁢C+ξ.

(3) S⁢G⁢O=b⁢0+b⁢1⁢P⁢C+b⁢2⁢S⁢i⁢z⁢e+b⁢3⁢M⁢a⁢t+b⁢4⁢C⁢r⁢e+b⁢5⁢M⁢C×P⁢C+ξ.

(4) S⁢G⁢O=c⁢0+c⁢1⁢P⁢C+c⁢2⁢S⁢i⁢z⁢e+c⁢3⁢M⁢a⁢t+c⁢4⁢C⁢r⁢e+c⁢5⁢M⁢C×F⁢C+ξ.

Equations 2, 3 add the interaction terms of government and heterogeneous stakeholder relationships to model (1). Based on model (1), Equation 4 adds the interaction terms of the market and financial relationships. When the coefficient of the interaction terms is greater than 0, the dual stakeholder relationship has a positive effect on social goal-oriented relationship. If the coefficient is significantly less than 0, the influence on social goal orientation from the perspective of the dual stakeholder relationship is weakened; if the coefficient is not significant, the influence is not obvious. This study standardizes and integrates different types of stakeholder relationships and conducts the corresponding empirical tests.

## Empirical tests

In this study, SPSS 24.0 was used for data processing, which controls the significance level based on a one-tailed test because the direction of the relationship in a pair of variables has already been specified. In order to ensure the consistency and validity of model estimation, data processing is as follows: (1) in order to overcome the influence of outliers, the main continuous variables were winsorized at the 1% level; and (2) in order to avoid the influence of multicollinearity, the interaction variables were centralized. In addition, all explanatory and control variables were diagnosed using the variance inflation factor (VIF), and the results showed that the VIF value was less than 2.0. Values significant at the 5% level are flagged with a single asterisk; those that are significant at the 1% level are flagged with two asterisks.

Since this study is a test of causation, there may be some unobservable factors that can both influence the strength of the government relationships and create barriers to new projects. Since these factors cannot be observed and have an impact on the explained variables, they cannot be added to the model as control variables. Finally, these unobservable factors related to government relationships are included in the error term, which leads to the endogeneity problem in the model (Sharma, 2019; Tian et al., 2019). In order to address the endogeneity problem, we divided the research framework into several sub-studies and carried out empirical tests for each sub-study.

### Descriptive statistics and relevant analysis

Table 3 reports the descriptive statistics of the main variables. According to the statistical results of the full sample, the mean value of (SGO) is 0.886, and the span between the maximum and minimum values is large, thus indicating high variance among SGO scores. The mean value of *FC* is 0.92, which indicates that having a financial relationship is common among listed companies in China, with 81.26% of enterprises having a financial relationship. The mean and standard deviation of *MC* are 0.55 and 0.497, respectively, which indicates that there is a large difference in market relationships, and the number of market relationships may have a strong correlation with the size of the enterprise and its credit rating. According to the results of the sub-sample description statistics, the mean value of SGO is 2.26, which preliminarily supports hypothesis 1. Other variables are in the normal range, and there are no extreme values. Table 3 also reports the correlation coefficients. Among them, SGO is positively related with government relationships, which indicates that social goal orientation in enterprises with a government relationship is higher than that in enterprises without one, which is consistent with hypothesis 1. Correlations between other variables are also reasonable. For example, the correlation coefficients between SGO and corporate maturity (*Mat*), credit rating (*Cre*), and government relationships (*PC*) are all significantly positive, which indicates that the higher the levels of debt and capital expenditures, the stronger the social goal orientations is. In addition, SGO is significantly positively correlated with *Mat*, which indicates that more mature enterprises have stronger social innovation preferences and higher social goal orientation.

**TABLE 3:** Descriptive statistics and correlation analysis.

Variable	Mean	SD	*PC*	*SGO*	*FC*	*MC*	*Cre*	*Mat*	VIF
*PC*	3.92	2.502	1						1.209
*SGO*	2.34	4.61	0.408***	1					
*FC*	2.56	1.982	0.305***	0.091***	1				1.219
*MC*	4.53	2.62	0.216***	0.093***	0.064***	1			1.438
*Cre*	48.28	19.69	0.253***	0.420***	0.305***	0.030**	1		1.011
*Mat*	25.36	4.541	0.084***	0.998**	0.064**	0.021	0.280***	1	1.142

*N* = 725. *t*-Statistics in parentheses; ***p < 0.001, **p < 0.01, *p < 0.05.

### Empirical test

Table 4 reports the regression results of model (1). The explained variable in columns (1) and (2) is *SGO*. Column (1) reports the regression results of the whole sample, and its explanatory variable is *PC*. Column (2) reports the regression results of the sample of enterprises with government relationships (i.e., all sample companies with government relationships are assigned a score of 1, 2, 3, or 4 according to their relationship strength; otherwise, they are assigned 0) whose explanatory variable is *PC*, which examines the influence between government relationships and SGO. The result shows that the number of companies that have government relationships is 725, and the regression results show that the regression coefficient of government relationships is significantly positive at the 1% significance level. In order to further test the linear relationship between government relationships and corporate social goal orientation, column (3) conducts an inverted U-shaped analysis of their correlation on the basis of column (2), and the results show that a government relationship cannot sustain and provide inexhaustible resources for long-term growth in social goal orientation. After the “vertex” effect between the government relationship and social goal orientation (β = 0.123, *p* < 0.01) is taken into consideration, the effect of government relationships on social goal orientation shows a decreasing utility pattern. This result supports hypothesis 1. Column (4) reports the regression results of the samples with financial relationships, which show that the correlation between financial relationships and corporate social goal orientation is not high, thus indicating that although financial relationships can relieve financing constraints, corporate social orientation across industries may be subject to financing discrimination and other problems. This result does not fully support hypothesis 2, and further analysis will be presented in the case of dual stakeholders. Column (5) reports the regression results of the sample of enterprises with market relationships, which shows that the regression coefficient of market relationships is significantly positive at the 1% significance level, thus indicating that the social goal orientation of companies with market relationships is higher than that of companies without. This result supports hypothesis 3. The regression results of the control variables are as follows. The estimated coefficient of corporate credit is significantly positive, which indicates that the higher the credit rating, the higher the social goal orientation will be, which is consistent with our expectations. The estimated coefficient of maturity is significantly positive, thus indicating that mature enterprises have higher social goal orientation, which is also in line with our expectations.

**TABLE 4:** Single stakeholder relationships → social goal-oriented regression analysis.

Variable	SGO				
	Model 1	Model 2	Model 3	Model 4	Model 5
*_cons*	28.078***	26.835***	25.283***	21.776***	24.109***
*Cre*	0.329*** (12.546)	0.193*** (9.987)	0.175*** (9.853)	0.209*** (12.745)	0.176*** (10.453)
*Mat*	0.064*** (2.709)	0.067** (2.783)	0.059* (2.653)	0.054* (2.339)	0.056* (2.479)
*PC*		0.183*** (9.233)	0.285*** (6.635)		
*PC × PC*			−0.143** (−2.754)		
*FC*				0.069* (2.750)	
*MC*					0.394*** (11.768)
*R* ^2^	0.054	0.081	0.076	0.057	0.093
Δ*R*^2^	0.041	0.069	0.063	0.049	0.080
*F*	77.357	80.650	62.293	53.225	97.643

Total *N* = 725. *t*-Statistics in parentheses; ***p < 0.001, **p < 0.01, *p < 0.05.

Tables 5, 6 report the probit regression results of models (2) and (3), respectively. Similarly, the explained variables in columns (1) and (2) are associated with social goal orientation, and the explanatory variables are government relationships and their interaction with *FC* and *MC*. We compare the differences in the influence of financial, market, and government relationships on social goal orientation. The results show that financial relationships with government relationships and that the interaction of regression coefficients was significantly positive. In addition, the results showed that when the CEO and board members worked for banking, securities, or other financial institutions, they are at the core of the network. That is, the corporate decision-makers have both government and financial relationships. Having the right social relationships can allow enterprises to make full use of their resources to advance their social goal orientation. The above results further verify hypothesis 4.

**TABLE 5:** Government–financial dual relationship regression analysis.

Variable	SGO		
	Model 1	Model 2	Model 3
*_cons*	29.674***	22.826***	18.376***
*Cre*	0.200*** (12.669)	0.120*** (9.173)	0.105*** (9.342)
*Mat*	0.040* (2.162)	0.046* (2.165)	0.015** (2.446)
*PC*		0.055*** (9.055)	0.103* (3.027)
*FC*		0.012 (1.356)	0.027*** (2.754)
*PC × FC*			0.453*** (4.352)
*R* ^2^	0.057	0.083	0.079
Δ*R*^2^	0.08	0.072	0.063
*F*	77.598	62.542	52.827

*N* = 725. *t*-Statistics in parentheses; ***p < 0.001, **p < 0.01, *p < 0.05.

**TABLE 6:** Government–market dual relationship regression analysis.

Variable	SGO		
	Model 1	Model 2	Model 3
*_cons*	29.241***	26.391***	25.085***
*Cre*	0.283*** (12.567)	0.194*** (9.684)	0.175*** (9.595)
*Mat*	0.074* (2.401)	0.031** (2.724)	0.041** (2.752)
*PC*		0.155*** (8.541)	0.114*** (3.402)
*MC*		0.150*** (3.854)	0.050 (1.204)
*PC × MC*			0.579* (2.583)
*R* ^2^	0.058	0.084	0.077
Δ*R*^2^	0.046	0.069	0.063
*F*	77.584	65.831	52.938

*N* = 725. *t*-Statistics in parentheses; ***p < 0.001, **p < 0.01, *p < 0.05.

Table 6 also reports the regression results of model (3), which explores the influence of the dual stakeholder relationship between the government and the market on social goal orientation. The regression coefficient of the interaction term shows that there is a marginally significant positive correlation between market and government relationships, which could be attributed to the follow three reasons. (1) During the market transformation process of Chinese enterprises, most homogeneous enterprises have a similar market relationships. (2) In order to maintain their market share and competitiveness, enterprises with strong market relationships are more inclined to use government relationships to obtain scarce resources and policy support, which could be used to secure venture investment in exchange for long-term returns. (3) The corporate cultural background in the Chinese context is based on relationship accumulation, but the relationship between the market and the government is always in a state of flux and the market factors that restrict growth differ from government factors (Luo and Lin, 2019). Therefore, the dual effect is lower than the single effect because the two relationships cancel each other out in terms of their.

This section empirically tests the role of the market–finance dual relationship on corporate social goal orientation and explores whether it is necessary for enterprises to have both. The results are shown in Table 7. The results show that there is no significant correlation between the finance–market dual relationship and corporate social goal orientation, which is different from the significant relationship between finance and single markets. The reasons are as follows. First, there is substitution between the market and financial relationships, but having both requires that enterprises have high cost and low return. Second, market relationships are not consistent across enterprises. The weak dual relationship between markets and finance may lead to higher financing constraints and information asymmetries due to the relationship between risk and returns, which mitigates corporate sociality. Therefore, the financial–market dual relationship is not necessary, but the influence of the government relationship on corporate social goal orientation is more significant than the financial–market dual relationship. The closer the correlation coefficient of *R* is to ±1, the stronger the correlation between the two variables is. It can be seen that all *R* values in Tables 4–7 are all greater than 0.09, which shows a correlation between the two variables given that all are greater than ±0.3. Moreover, there is no significant difference between the adjusted *R*^2^ and *R*^2^, which means that the independent variables used can clearly measure the changes in the dependent variables. However, Δ*R*^2^ has a small gap with *R*^2^, which indicates that the model fit is relatively stable. Nevertheless, the Δ*R*^2^ value of the main effect is less than 0.5. In the regression analysis, 0.5 is the critical value of the adjusted *R*^2^. If the adjusted *R*^2^ is less than 0.5, the explanatory power of the model is weak. However, in this study, when exploring the correlation between stakeholder relationships and corporate social goal orientation, other variables that have shown strong explanatory power in previous studies have been incorporated into the model as control variables. Based on the previous research results, this study identifies a new interpretable result. Although the explanatory power of this model is low, it is relatively stable. Moreover, the robustness test of the model carried out in Table 8 also confirms that the model is relatively stable although its strength is insufficient.

**TABLE 7:** Financial–market dual relationship regression analysis.

Variable	SGO		
	Model 1	Model 2	Model 3
*_cons*	29.241***	19.833***	16.044***
*Cre*	0.276*** (12.844)	0.219*** (9.984)	0.193*** (9.384)
*Mat*	0.074* (2.106)	0.031** (2.884)	0.041* (2.257)
*FC*		0.011 (1.139)	0.074* (1.228)
*MC*		0.156*** (11.039)	0.225*** (4.603)
*FC × MC*			0.087 (1.674)
*R* ^2^	0.69	0.077	0.096
Δ*R*^2^	0.056	0.063	0.079
*F*	77.769	73.641	59.414

*N* = 725. *t*-Statistics in parentheses; ***p < 0.001, **p < 0.01, *p < 0.05.

**TABLE 8:** Robustness test.

Variable	SGO_1_		
	Model 1	Model 2	Model 3
*_cons*	21.241***	16.487***	14.469***
*Cre*_1_	0.265*** (9.523)	0.349*** (6.579)	0.326*** (6.537)
*Mat*_1_	0.237* (2.568)	0.027* (0.783)	0.042 (1.299)
*PC*_1_		0.164*** (6.797)	0.110* (2.093)
*FC*_1_		0.023* (1.672)	0.132*** (3.318)
*MC*_1_		0.128*** (8.485)	0.592*** (3.880)
*PC*_1_ *× MC*_1_			0.469*** (6.690)
*PC*_1_ *× FC*_1_			0.405** (3.137)
*MC*_1_ *× FC*_1_			0.080 (1.517)
*R* ^2^	0.067	0.094	0.097
Δ*R*^2^	0.056	0.082	0.083
*F*	47.633	45.982	35.819

*N* = 725. *t*-Statistics in parentheses; ***p < 0.001, **p < 0.01, *p < 0.05.

### Robustness test

The explanatory variables in model (1) were calculated using data from 2009 to 2020. In order to test the stability of the results, the data were replaced with the sample data from 2013 to 2020, which we obtained using standardized calculation. We then re-run model (1) using the substitute variables. In addition, in order to verify the robustness of hypotheses 4–6, we perform group tests on models (2) and (3). Given our hypothesis on the effect of the external corporate environment on stakeholder relationships, the grouping tests can be divided into three categories (i.e., government, financial, and the market relationships). In order to avoid interference between heterogeneous stakeholder relationships, the data were classified by group and standardized to test hypothesis 2. The results show that hypotheses 1–3 are significantly positive at the 1% significance level, which further demonstrates that stakeholder relationships are conducive to promoting corporate social goal orientation, and dual stakeholder relationships have a stronger effect than single-stakeholder relationships. Therefore, the results further support hypotheses 4 and 5, and hypothesis 6 remains consistent with the original results.

## Discussion

Based on the data disclosed by a 4288 sample of 725 Chinese-listed companies, this study constructed a model of the relationships between stakeholder relationships and corporate social goal orientation. The results show that based on the resource dependence theory. We find an inverted U-shaped relation between government relationships and social goal orientation. However, the relationships between different types of stakeholders and social goal orientation have heterogeneous effects. First, both the financial and the market relationships have a positive influence on social goal orientation. However, the influence of the market–government dual relationship on social goal orientation is positive and significant. Last, the influence of the finance–government dual relationship on social goal orientation is more significant than that of the single stakeholder relationships, but there is no significant correlation between the market–finance dual relationship and corporate social goal orientation.

The results verify the importance of stakeholder relationships to social development in the context of companies promoting of their own social values. From the perspective of enterprises, government relationships not only bring the capital needed for social development, but also provide new political opportunities and form an informal feedback channel between enterprises and the government, which is conducive to the “transmission” of corporates’ social innovation policies (Liu et al., 2017). In addition, the close relationships between enterprises, banks, and other financial institutions also provide an implicit guarantee for the financial resources required to achieve social goals. The market uses the “invisible hand” to regulate participation and competition (Li and Wang, 2015; Chen Y. et al., 2018) which highlights the importance of making and sustaining connections.

However, seeking political resources to reduce operational uncertainty has become the first choice of most enterprises, and the government is willing to cooperate with mature companies with an established market presence (Chen et al., 2018). It follows that access to government resources and enhancing relationships with government officials is an integral part of corporate strategic decision-making. Effectively accessing resources can be summarized by the following three points. First, enterprises that have been recognized as high-tech enterprises can receive preferential policy treatment. Second, enterprises can enhance their reputation and improve their social influence. Third, enterprises can take the initiative in pursuing CSR. Therefore, government relationships allow the government to influence social development through informal means and effectively control social goal-oriented trends (Jin et al., 2016). How stakeholder relationships drive the use of resources is indeed a key factor that affects social goal orientation, but a lack of or imbalance in resources will also pose resource risks. The imbalance of a resource poses a potential threat to corporate social goal orientation in general. Access to government resources is thus an integral part of corporate strategic decision-making.

## Conclusion

The confirmation of these hypotheses enables us to draw a conclusion about the importance of stakeholder relations to social goal-oriented development at the stage when enterprises enhance their social value. In recent years, the field has developed rapidly at the economic-social level. Many researches based on China’s capital market also show that when going deep into the internal operation of small and micro enterprises, CSR are more reflected as the value weapon to enhance shareholder wealth (Gao et al., 2020). However, in the past, the performance of CSR was to consider the impact of corporate behavior on society from the perspective of the whole society, and was concerned about the relationship between enterprises and society (Carroll, 2021). As the ultimate goal of corporate socialization, scholars believe that corporate social goal orientation ultimately lies in the satisfaction of interests, and corporate operation is the coexistence and dynamic change of multiple subjects (Farmaki, 2019). Therefore, corporate social goal orientation has an inseparable dependency relationship with various subjects in the external system, and the establishment of external core stakeholder relations is a key factor conducive to the enterprise’s own development as well as the effective allocation of scarce social resources. Second, maintaining a positive relationship quality with core stakeholders such as society, meeting their needs, and solving social problems, such as employment, entrepreneurship, and pension are conducive to market and social integration (Barba-Sánchez et al., 2021). Although some studies have explored innovation-driven corporate value from the perspective of profitability (Jin et al., 2016), they have ignored the dynamics of corporate social goal orientation behaviors at the micro-level and how stakeholder relationships promote its development. Therefore, in this article, we focuses on the driving factors of corporate social goal orientation and attempts to analyze the interaction between informal institutions and social goal orientation at the micro-level (Ionescu, 2021). At the same time, we introduce stakeholder relationships into the research on corporate social goal orientation, thereby providing a new perspective to the literature. By taking the informal relationships between enterprises and external systems as the research topic, this study fills the existing research gap of social goal orientation in the context of stakeholder relationships. Furthermore, this research constructs a “stakeholder relationships–social goal orientation” model and clarifies the nature of the relationships between the government, the financial sector, and the market. Thus, this study deepens our understanding of entrepreneurs’ psychology and decision-making in the context of social goal-oriented development. Finally, in practice, the development of corporate social goal orientation is still insufficient. That is, because of the reform created by the “government-enterprise-society” tripartite structure, various stakeholder groups have been greatly impacted, but no credible changes have been suggested (Kujala et al., 2022). The subject of social goal orientation encourages and implements certain behaviors, which highlights the role of stakeholders and their values in advancing social goal orientation (Barba-Sánchez et al., 2021). Therefore, this study recommends governance strategies and policy suggestions for solving social problems, meeting social demands, and developing social value from the multiple-stakeholder relationship perspective.

However, due to objective limitations, the study of social goal orientation and effectiveness in this article is only a beginning, and there are still many limitations: First, the study is based on the socio-economic framework, so it only considers the relationship between major stakeholders in the external environment. However, internal stakeholders undoubtedly have a certain influence. For example, the annual entertainment expense of an enterprise is also an indicator of the strength of its social relationship. Second, it is difficult to avoid the sensitivity of stakeholder relationships in building a complete system that relies on social orientation to generate profits. The transition from commercial profitability to social orientation is not binary, but rather a continuous development process. Therefore, enhancing social goal orientation will be a gradual process. In the future, the proposed model can be tested in different contexts to obtain results that are more detailed. Furthermore, enterprises are in the process of transforming into being more socially oriented actors (Barba-Sánchez et al., 2021). Whether the social goal-oriented model proposed in this article will change the final enterprise model must undergo long-term observation and further testing through longitudinal data. Finally, Future research should examine both the economic and social value of enterprises and analyze how stakeholders pursue sustainable development in different systematic contexts.

## Data availability statement

The data analyzed in this study is subject to the following licenses/restrictions: The database involves private data of Chinese listed companies, and datasets is restricted. Requests to access these datasets should be directed to https://www.gtarsc.com/.

## Author contributions

XL and YS: conceptualization, data curation, and methodology. XL: formal analysis, resources, software, and writing—original draft. YS: funding acquisition, project administration, and supervision. YX: validation. XL, YS, YX, and WW: writing—review and editing. All authors contributed to the article and approved the submitted version.

## Footnotes

1 Rankins CSR Ratings (RKS) is an authoritative third-party rating agency of corporate social responsibility in China. It is committed to providing objective and scientific corporate responsibility rating information for SRI, responsible consumers and the public. RKS includes ESG (i.e., environment, social responsibility, and governance) ratings, CSR ratings, CSR investor services, etc.

2 Cninf is the information disclosure website of listed companies designated by the China Securities Regulatory Commission.
